# Long-term consequences of COVID-19 on mental health and the impact of a physically active lifestyle: a narrative review

**DOI:** 10.1186/s12991-023-00448-z

**Published:** 2023-05-11

**Authors:** Leonardo Roever, Bruno Raphael Ribeiro Cavalcante, Alex Cleber Improta-Caria

**Affiliations:** 1 Department of Clinical Research, Federal University of Uberlândia, Uberlândia, Brazil; 2 Gonçalo Moniz Institute, Oswaldo Cruz Foundation (IGM-FIOCRUZ/BA), Salvador, Brazil; 3 Department of Pathology and Forensic Medicine, School of Medicine, Federal University of Bahia (UFBA), Salvador, Brazil; 4 Post-Graduate Program in Medicine and Health, Faculty of Medicine, Federal University of Bahia (UFBA), Salvador, Brazil

**Keywords:** Coronavirus, Physical activity, Physical exercise, Memory, Anxiety, Depression

## Abstract

**Background:**

Coronavirus-19 disease (COVID-19) is caused by the severe acute respiratory syndrome coronavirus 2 (SARS-CoV-2). Respiratory viruses damage not only the upper respiratory tract in humans, but also several different organs such as the brain. Some of the neurological consequences of COVID-19 reported are anosmia, headache, stroke, declined cognitive function, and impaired mental health, among others. People who had COVID-19 have a higher risk of sequelae in the central nervous system (CNS). However, it is not known which are all possible sequelae and how long will last the long-term effects of the COVID-19 pandemic on behavioral patterns and quality of life.

**Aim:**

We intend to address the long-term impacts of COVID-19 on mental health and the relevance of physical exercise during the pandemic.

**Methods:**

We conducted a literature search using PubMed to find the articles that were related to these themes.

**Results:**

We found 23,489 papers initially, and then we applied the inclusion/exclusion criteria to narrow down our search to 3617 articles and selected 1380 eligible articles after a thorough reading of titles and abstracts. The findings indicated that COVID-19 impacted general mental health and led many not only hospitalized patients to develop cognitive decline, memory impairment, anxiety, sleep alterations, and depressive-like behavior. Furthermore, the fear of vaccines and their effects had negatively affected mental health and directly impacted mortality rates in unvaccinated COVID-19 patients.

**Conclusions:**

Preventive measures must be undertaken, such as the vaccination of the entire population, vaccination hesitancy discouragement by creating awareness among individuals, and people’s engagement in a physically active lifestyle, since being physically active is a low-cost and effective measure to restore or inhibit the negative outcomes from COVID-19 on mental health.

## Introduction

Severe acute respiratory syndrome coronavirus 2 (SARS-CoV-2) is a virus that promotes the coronavirus-19 disease (COVID-19), which has killed millions of people worldwide [52]. Respiratory viruses are pathogens that will infect mainly the upper respiratory tract in humans, but also damage several different organs such as the liver, kidneys and brain [52, 88]. A body of evidence of SARS-CoV-2 detrimental effects on the brain is still emerging, but, based on the similarities between SARS-CoV-2 and other coronaviruses it is expected that changes in endocrine and immune response in the periphery or in the central nervous system (CNS) might occur contributing on this way to inflammation, oxidative stress and impaired mental health [73, 91].

SARS-CoV-2 can invade the brain through several different pathways like through infected neurons that connect the periphery to the brain (i.e., motor neurons), or it may enter through the olfactory nerve, or the virus could even cross the blood–brain barrier [88, 91]. The outcome varies depending on each individual, however, some of the most common neurological consequences of COVID-19 reported until now are anosmia, headache, stroke, impaired consciousness, seizures, coma, stroke, encephalopathy, declined cognitive function, and impaired mental health [50, 61, 63].

Part of the acute and long-term effects on the CNS can be explained by the inflammation caused by COVID-19, which has been shown to play a crucial role in CNS, especially when induced by a cytokine storm [24, 50]. This term refers to a life-threatening systemic inflammatory syndrome related to elevated levels of circulating cytokines and immune-cell hyperactivation that can be triggered by various therapies, monogenic disorders, cancers, pathogens, and autoimmune conditions [40]. As a matter of fact, individuals with underlying conditions that cause an immunocompromised state are considered vulnerable to coronavirus infection. For instance, in systemic lupus erythematosus patients, the use of systemic glucocorticoids and immunosuppressants, are potential susceptibility factors when considering the risk of contracting COVID-19 and infection outcomes [41]. Likewise, patients with myasthenia gravis, a chronic autoimmune neuromuscular disease, often treated with immunosuppressants, might be at higher risk of developing COVID-19, possible respiratory failure when concomitant with the infection, and demonstrating a severe disease course [9]. In summary, while the antiviral immune response is important to confer protection from coronavirus infection, an uncontrolled proinflammatory cytokine response can lead to a cytokine storm that compromises the lungs’ and other organs’ functions, and will ultimately cause significant morbidity and mortality, especially in immunocompromised individuals.

Apart from the immunological features of SARS-CoV-2 on the CNS, there are the long-term mental and physical health consequences of COVID-19, which can become a major public health concern. Many countries were forced to adopt restrictive measures, including social distancing or total isolation protocols for their populations, which were shown to be the most effective measures to inhibit the fast dissemination of the SARS-CoV-2 [14, 55]. If, on the one hand, these measures proved to be successful in controlling viral spread, on the other hand, it had presented a greater impact on public mental health [15, 74]. Indeed, the social isolation period during COVID-19 outbreak deeply affected the quality of life, including a reduction in physical activity daily routine, contributing to the development and progression of mental disorders, such as cognitive decline, memory impairment, depression and anxiety [15, 56, 74]. In addition, it is noteworthy that the virus transmission is still expected to happen on a large scale until 2024, and intermittent social distancing or total isolation may occur again impacting greatly the lifestyle of the world population [55, 56]. Furthermore, people who had COVID-19 presented higher risk of death and sequelae in the respiratory system and CNS that could lead to the development of neurocognitive disorders and mental health disorders 30 days after halting the symptoms of the disease [1, 81].

In summary, prospective cohort studies associating COVID-19 and mental health are still rising, so causal implications between the presence of mental disorders and COVID-19 severity or morbidity are limited. In this context, there is an imperative need to update the mental health status in light of new findings since there are some gaps in the literature regarding the long-term effects of the COVID-19 pandemic on behavior patterns and quality of life once life returns to normal in the entire world. Therefore, the rationale of this review addresses the impact of the COVID-19 pandemic on general mental health suggesting preventive measures that should be undertaken to avoid long-term negative outcomes and how having a physically active lifestyle can be beneficial in this matter.

## Methods

### Search strategy

PubMed/Medline search strategy was used in this narrative review. Our search strategy combined Medical Subject Headings (MeSH) terms, their relevant synonyms, and the Boolean operators “AND” and “OR”. Concept clusters were included: (1) COVID-19, (2) mental health, (3) depression (4) anxiety, (5) memory impairment, (6) cognition, (7) cognitive decline, (8) lifestyle, (9) physical exercise, (10) exercise, (11) physical activity, (12) SARS-CoV-2, and (13) pandemic.

The search was performed, and results were exported in Comma-Separated Values (CSV) format to Microsoft Excel. Subsequently, duplicates were removed using their PubMed ID and title and then proceeded with the inclusion and exclusion of the studies.

## COVID-19 pandemic impacts on cognitive function and memory

COVID-19 might lead to a decline in cognitive function and memory impairment, the development of psychoses, and many different symptoms related to posttraumatic stress disorder leading to the impairment in neuronal survival and growth, synaptic plasticity, and reduction in the production and release of neurotransmitters [73, 91]. People who develop severe COVID-19 and survive often complain about having problems with cognition and memory [2]. A recent study evaluated 740 people with an average age of 49 years old displaying a median time between presenting symptoms and mental evaluation of 7 months [4]. This study showed that among the most notable neurological issues patients presented memory deficits (24%) mainly related to memory recall and memory speed.

Another recent study did a systematic review about how COVID-19 would affect cognition and memory and found six studies documented the prevalence of cognitive and memory impairment, and one that quantified the deficits after recovery [2]. The authors estimated cognitive impairment ranging from 43.0 to 66.8% prevalence in all hospitalized COVID-19 patients. These numbers are quite high and preventive actions such as vaccinating the population could have reduced the number of severe cases and the risks of developing cognitive deficits and memory impairment. It has been reported that people who have had COVID-19 can also present a greater loss of grey matter in the brain [38].

Douaud et al. investigated brain changes in 785 UK Biobank participants aging 51 to 81 years old [38]. The most important approach in this study is the fact that the brain images were taken before and after the SARS-CoV-2 infection. This action eliminates any biases from pre-existing brain structure changes. The authors identified a significant reduction in grey matter thickness and tissue contrast in the hippocampal gyrus and orbitofrontal cortex, which are essential areas of the brain for the normal functioning of cognitive function and memory. Then, the authors further suggested that the loss of grey matter in brain regions related to cognitive function and memory can dramatically increase the risk of inflammation, reactive gliosis, and developing dementia, such as Alzheimer`s disease [23]. It was also identified greater changes in markers of tissue damage in brain areas related to olfactory cortex, and a significant reduction in global brain size with the participants showing cognitive decline between the 2 time points. These findings together may explain the persistent problems related to loss of smell and memory issues often seen in patients who recovered from COVID-19. Whether this effect will persist in the long-term and if these deleterious impacts can be partially reversed is not known yet. Nevertheless, the loss of grey matter in people who were infected by COVID-19 can deeply impact cognition and lead to mental disorders, such as anxiety and depression.

## COVID-19 pandemic impacts on sleep, anxiety, and depression

A recent study revealed that racial, social, and economic differences impacted mental health in older adults during COVID-19 pandemic [85]. The study showed that black individuals showed significantly greater posttraumatic growth compared to non-Hispanic, and White participants. Even when controlling the effects of stress and coping strategies, race kept being significantly associated with posttraumatic growth. Thus, COVID-19 pandemic has disproportionately affected people who are more vulnerable in both physical and mental health. Another recent study linked Israeli national databases (COVID-19 testing, infection, hospitalization, mortality, and vaccinations with all subjects ever hospitalized for a psychiatric disorder) to the total population [45]. The authors showed the need to increase testing for COVID-19 in individuals ever hospitalized for a psychiatric disorder, as their ratio attributed mortality and all-cause mortality were much higher than the cases in the total population.

COVID-19 pandemic has triggered the alarm for the individual and collective importance of mental health and social functioning [70]. The most commonly known mental disorders are anxiety and depression, which present a variety of prevalence rates [32, 86]. The development and progression of an anxious profile and a depressive-like behavior might induce a negative effect on several dimensions of quality of life [21, 83]. One of the first studies to evaluate the impacts of COVID-19 on these mental disorders was done in China on February 20th-2020, where the authors surveyed 369 adults in 64 cities revealing that 27% of the participants worked at the office, 38% resorted to working from home, and 25% stopped working due to the outbreak [90]. It was reported in those who stopped working during the pandemic presented a decline on mental and physical health conditions as well as distress. In addition, a longitudinal study focusing on older adults' mental health during the COVID-19 pandemic in Argentina showed that the mental health trajectory had a complex and heterogeneous dynamic. Indeed, it was shown that individuals that were younger, females, presented pre-existing psychiatric diagnoses, or had lower resilience were more likely to experience fluctuations in psychological distress [42].

It is noteworthy that sex and gender are important modifiers of mental health in normal times and during crises [26, 27, 29, 32, 33]. After the onset of the COVID-19 pandemic, health risks and outcomes disproportionately affected women, making them a vulnerable group due to multiple stresses and mental health symptoms [79]. This can be explained by the fact that gender roles tend to be reinforced during times of disease outbreak, which likely leads to feelings of stress, dissatisfaction, and lack of autonomy.

As social isolation was an effective measure against the virus adopted in many countries worldwide, it is expected that millions of people had substantial impact on their physical and mental health conditions. A systematic review and meta-analysis evaluated the prevalence of stress, anxiety and depression in the general population during the COVID-19 pandemic and showed that there was a prevalence of developing an anxious profile in 31.9% of the studies, while the prevalence of depression was 33.7% of the total number of studies [74]. During COVID-19 pandemic the adoption of a sedentary behavior was also often seen due to quarantine and social isolation and these factors combined leaded many people to gain weight or even to develop obesity, which is intrinsically associated with memory impairment in humans and in different animal models of obesity [7, 33].

Sleep and the circadian system exert a strong regulatory influence on immune functions. Indeed, it has become increasingly clear that sleep and the immune system are bidirectionally linked, as immune system activation alters sleep, and sleep, in turn, affects the innate and adaptive immune system [6]. Sleep deprivation may lead to a chronic inflammatory state, an increased risk for infectious/inflammatory pathologies, such as autoimmune and neurodegenerative diseases, and a negative impact on mental health, favoring anxiety and depression conditions [16, 43]. Many people have experienced sleeping difficulties, and the crosslink between impaired sleep efficiency and disrupted innate immunity makes people susceptible to viral infections [65]. A recent meta-analysis suggested that 45% of COVID-19 patients experience depression, 47% of patients experience anxiety, and 34% of patients experience sleep disturbances, considering a majority of Chinese patients [34].

It is well known that being physically active can inhibit the development or progression of an anxious and depressive-like behavior [19]. Therefore, the adoption of a healthy lifestyle, mainly through the regular practice of physical exercise, has been extensively recommended as an effective and low-cost non-pharmacological approach to minimize the consequences of social distancing/isolation during COVID-19 pandemic [17]. Nevertheless, due to the long-term consequences of COVID-19 on general mental health the adoption of a physically active routine is a must and a preventive measure that can save millions to public safes.

## COVID-19 pandemic and the importance of engaging in a physical exercise routine

Physical exercise can be divided mainly into 2 different types: aerobic exercise and resistance exercise [25, 75]. Aerobic exercise consumes higher levels of oxygen and, predominantly, recruits red skeletal muscle fibers [44, 71], while resistance exercise recruits more white fibers and is mainly focused on developing strength [28, 31, 76]. It is well established that engaging in a physical exercise routine will reduce the risk of developing cardiovascular diseases, diabetes, obesity, and several other chronic diseases [35, 59, 69]. Engaging in a physically active lifestyle will also induce beneficial effects on the brain, such as a greater blood flow to the hippocampus e prefrontal cortex, and will provide enhancement in the number of synapses, consequently, favoring neurogenesis, and these physiological changes will lead to improvement in cognitive function, memory and mental health [12, 28, 87].

COVID-19 pandemic-associated quarantine has taken the population to develop or enhance a sedentary lifestyle [58]. Results from a multicenter observational study estimated the impact of COVID-19 restrictions on migraine severity during 2020 March–May lockdown in Italy showing that 28% of patients reported a headache worsening, 33% an improvement, and 39% a stable headache frequency alongside a significant decrease of the physical activity levels [36]. Also, the COVID-19 pandemic had the potential to severely affect patients with neuromuscular disorders (NMD) as infection commonly triggers exacerbation or progression of NMD [47]. Therefore, increased rates of disease worsening can be expected during the COVID-19 pandemic although data regarding this topic are still lacking. Nonetheless, the pandemic may be related to mental health challenges for people living with multiple sclerosis due to social interaction restrictions, interrupted routines, and reduced options for safe physical exercise [18]. Likewise, a study from India regarding patients with myasthenia gravis showed that COVID-19 and lockdown have been associated with poor quality of life, impaired activity of daily living, and poor sleep quality [53]. Altogether, these results gave a glimpse of how the restrictions taken during the pandemic have affected the practice of physical activity levels, sleep quality, and mental health.

There is a growing body of evidence indicating that aerobic and resistance training has positive effects on physiological and mental health in humans [11, 20, 29] and in several different animal models [31, 54]. It has been recently reported that engaging in regular physical activity can postpone the onset of a first-ever stroke and improves long-term outcomes in the elderly [64], what is extremely important once COVID-19 had a greater impact on this population. Changing lifestyle habits through engaging in a physically active routine is an excellent and effective therapy against the impacts of COVID-19 on physical and mental health and in many other diseases, such as cardiovascular and neurodegenerative diseases [10, 13, 27, 49]. Therefore, it is urgent the development of strategies to engage the population in a physically active routine to halt the impacts of COVID-19 social measures on mental health.

## Vaccination hesitancy and mental health during the COVID-19 pandemic

Vaccination is a game changer to limit the pandemic spread of SARS-CoV-2/COVID-19, apart from being the most cost-effective public health strategy. However, vaccines can only be successful if the overall population show acceptance to get vaccinated, which is not entirely seen when concerning proportions of populations worldwide show vaccine hesitancy. The fear of vaccines and their effects have negatively affected mental health, which directly impacts mortality rates in unvaccinated COVID-19 patients.

Some factors may explain the occurrences of vaccine hesitancy, such as premature approval without long-term safety data, potential adverse effects, and confidence about the vaccine’s efficacy before administration [68]. To make things worse, the spread of misinformation regarding the side effects and the conspiratorial theories related to the newer generation vaccines led the general public to become either skeptical or confused and anxious about whether to get vaccinated or not. It is worth mentioning that the unvaccinated people around the world might present a greater risk for the emergence of new variants of concern, such as Omicron, and vaccination hesitancy may provide a solid ground for this scenario.

In summary, to tackle COVID-19 pandemic negative effects and improve population mental health it should be given emphasis to reduce vaccination fear by creating awareness among the people and promote a positive social attitude.

## COVID-19 pandemic effects on mental health and the molecular mechanisms linked to exercise and mental health

When talking about the adherence to physical exercise and its impact on mental health during the pandemic is important to evaluate and compare individuals who are meant to be healthy with the ones who already have any chronic disease. A recent systematic review and meta-analysis evaluated the differential impacts of COVID-19 pandemic on physical activity involvement and exercise habits in people with and without chronic diseases [66]. The authors reported that objective and self-reported assessments conducted during the pandemic revealed a significant reduction in the levels of physical activity in both people with or without chronic diseases. Furthermore, the analysis revealed low levels of engagement in a physically active routine to be linked to countries where lockdowns were made, what is expected as social isolation will influence daily routine.

Anxiety and depression promote epigenetic changes [57, 78] and modifications in several molecular signaling pathways [37, 62]. One of the main hallmarks associated with the development of depressive symptoms is endothelial dysfunction [82]. Decreased levels of the myokine irisin are associated not only changes in adipose tissue [8, 22], but also in endothelial dysfunction and the development of a depressive-like behavior [80]. Adequate immune system response is also dependent on the regular practice of physical exercise. It is known that physical exercise can increase irisin expression [5, 51] and improve endothelial function [10] inducing neuronal survival to contribute to a better mental health [26].

One of the major altered pathways in mood disorders is the phosphatidylinositol 3 kinase (PI3K)/protein kinase B (AKT) pathway [72]. The development of an anxious profile or a depressive-like behavior can induce the inhibition of PI3K expression with a consequent decrease in the PI3K/AKT pathway, which is associated with a reduction in cell proliferation and survival and, conversely will lead to increased expression of glycogen synthase kinase 3 beta (GSK3β), a marker of neuronal death. Engaging aerobic exercise routine promotes increased expression of PI3K and activation of the PI3K/AKT signaling pathway, increasing cell proliferation and survival and decreasing neuronal death [84].

**Fig. 1:**
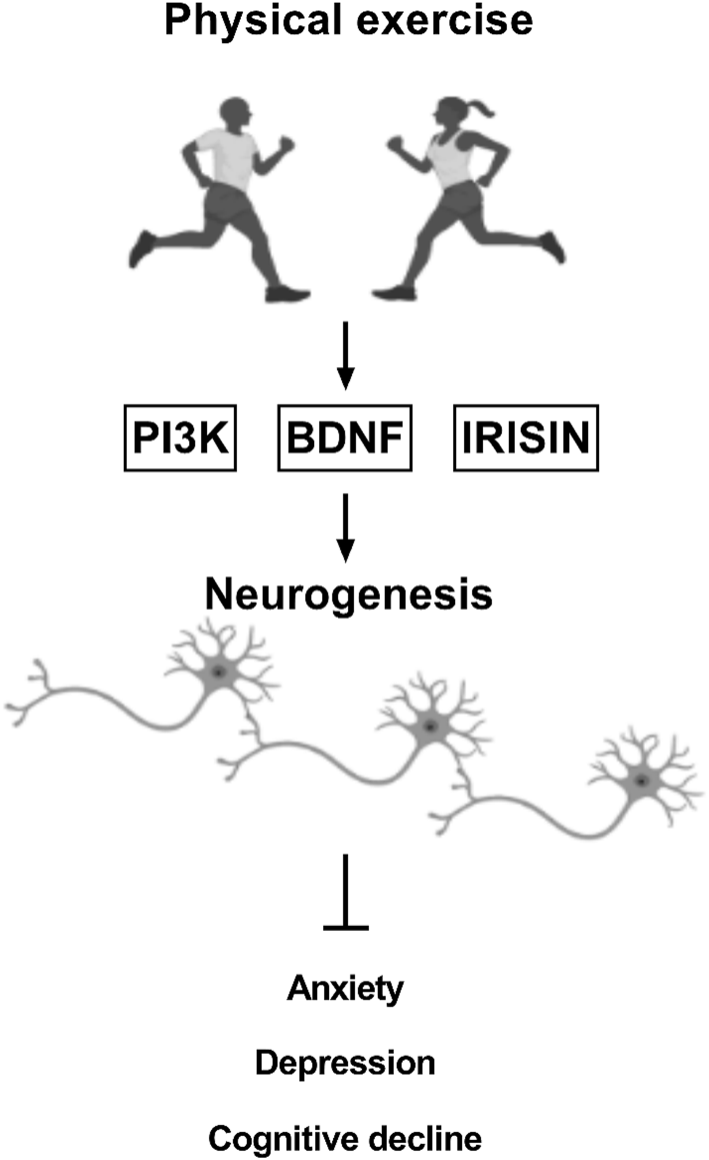
Physical exercise improves general mental health through activating different signaling pathways. Engaging in a physically active routine will induce the activation of PI3K pathway, enhance BDNF and irisin levels, favoring neurogenesis and inhibiting the development of an anxious profile, a depressive-like behavior and cognitive decline

Another well-known mechanism that occurs in mood disorders and cognitive decline is the reduction of brain-derived neurotrophic factor (BDNF) levels, inducing neuronal plasticity dysfunction [60] and decreased neurogenesis [89]. However, performing physical exercise on a regular basis will generate an increase in the β-hydroxybutyrate metabolite that inhibits class I histone deacetylases (HDACs) that will activate BDNF promoters [39], leading to increased neurogenesis, improving cognitive function and alleviating anxiety and depression symptomatology (Fig. 1).

It is known that engaging in physical activity will not only avoid developing cognitive decline and memory impairment [3], but also will fight the development and progression of obesity [48], type 2 diabetes [67], and many other related diseases. Finally, investigating molecular alterations through physical exercise interventions can also provide an interesting and cheap approach to evaluating changes in health biomarkers [30, 46].

## Limitations

This narrative review lacks assessment of publication bias with concomitant detailed intervention and main outcomes of each study used throughout this text, which can be viewed as its main limitations. It is also missing a more detailed study methodology using a higher number of databases, presenting inclusion and exclusion criteria and a more detailed study selection approach.

## Conclusions

Social distance and total isolation due to COVID-19 pandemic impacted negatively on mental health. Many patients developed an anxious profile, and a depressive-like behavior. People who got the virus and developed more severe symptoms of COVID-19 and survived often have memory issues that impact cognitive function and daily activities. These are long-term predicted changes in general mental health due to COVID-19 pandemic and preventive measures must be undertaken, such as the vaccination of the entire population and stimulating people to engage in a physically active lifestyle. Being physically active is a low-cost and effective measure to restore or inhibit the negative outcomes of COVID-19.

## Data Availability

Not applicable.

## References

[1] Al-Aly Z, Xie Y, Bowe B (2021). High-dimensional characterization of post-acute sequelae of COVID-19. Nature.

[2] Alnefeesi Y, Siegel A, Lui LMW (2021). Impact of SARS-CoV-2 infection on cognitive function: a systematic review. Front Psychiatry.

[3] Batmyagmar D, Kundi M, Ponocny-Seliger E (2019). High intensity endurance training is associated with better quality of life, but not with improved cognitive functions in elderly marathon runners. Sci Rep.

[4] Becker JH, Lin JJ, Doernberg M (2021). Assessment of cognitive function in patients after COVID-19 infection. JAMA Netw Open.

[5] Benedini S, Dozio E, Invernizzi PL (2017). Irisin: a potential link between physical exercise and metabolism - an observational study in differently trained subjects, from elite athletes to sedentary people. J Diabetes Res.

[6] Besedovsky L, Lange T, Haack M (2019). The sleep-immune crosstalk in health and disease. Physiol Rev.

[7] Boidin M, Handfield N, Ribeiro PAB (2020). Obese but fit: the benefits of fitness on cognition in obese older adults. Can J Cardiol.

[8] Boström P, Wu J, Jedrychowski MP (2012). A PGC1-α-dependent myokine that drives brown-fat-like development of white fat and thermogenesis. Nature.

[9] Businaro P, Vaghi G, Marchioni E (2021). COVID-19 in patients with myasthenia gravis: epidemiology and disease course. Muscle Nerve.

[10] Caria ACI, Nonaka CKV, Pereira CS (2018). Exercise training-induced changes in microRNAs: beneficial regulatory effects in hypertension, type 2 diabetes, and obesity. Int J Mol Sci.

[11] Cassilhas RC, De Sousa RAL, Caxa L (2022). Indoor aerobic exercise reduces exposure to pollution, improves cognitive function, and enhances BDNF levels in the elderly. Air Qual Atmos Heal.

[12] Cassilhas RC, Lee KS, Fernandes J (2012). Spatial memory is improved by aerobic and resistance exercise through divergent molecular mechanisms. Neuroscience.

[13] Cavalcante BRR, Improta-Caria AC, de Melo VH, De Sousa RAL (2021). Exercise-linked consequences on epilepsy. Epilepsy Behav.

[14] Chang SL, Harding N, Zachreson C (2020). Modelling transmission and control of the COVID-19 pandemic in Australia. Nat Commun.

[15] Chou SHY, Beghi E, Helbok R (2021). Global incidence of neurological manifestations among patients hospitalized with COVID-19—a report for the GCS-NeuroCOVID consortium and the ENERGY consortium. JAMA Netw Open.

[16] Clement-Carbonell V, Portilla-Tamarit I, Rubio-Aparicio M, Madrid-Valero JJ (2021). Sleep quality, mental and physical health: a differential relationship. Int J Environ Res Public Health.

[17] da Silveira MP, da Silva Fagundes KK, Bizuti MR (2021). Physical exercise as a tool to help the immune system against COVID-19: an integrative review of the current literature. Clin Exp Med.

[18] Davis BE, Lakin L, Binns CC (2021). Patient and provider insights into the impact of multiple sclerosis on mental health: a narrative review. Neurol Ther.

[19] de Lima NS, De Sousa RAL, Amorim FT (2022). Moderate-intensity continuous training and high-intensity interval training improve cognition, and BDNF levels of middle-aged overweight men. Metab Brain Dis.

[20] De Melo VH, De Sousa RAL, Improta-Caria AC, Nunes MAP (2021). Physical activity and quality of life in adults and elderly individuals with lower limb amputation. Rev Assoc Med Bras.

[21] de Oliveira LRS, Machado FSM, Rocha-Dias I (2022). An overview of the molecular and physiological antidepressant mechanisms of physical exercise in animal models of depression. Mol Biol Rep.

[22] de Oliveira M, De Sibio MT, Mathias LS (2020). Irisin modulates genes associated with severe coronavirus disease (COVID-19) outcome in human subcutaneous adipocytes cell culture. Mol Cell Endocrinol.

[23] De Sousa RAL (2022). Reactive gliosis in Alzheimer’s disease: a crucial role for cognitive impairment and memory loss. Metab Brain Dis.

[24] De Sousa RAL (2019). Gestational diabetes is associated to the development of brain insulin resistance in the offspring. Int J Diabetes Dev Ctries.

[25] De Sousa RAL (2018). Brief report of the effects of the aerobic, resistance, and high-intensity interval training in type 2 diabetes mellitus individuals. Int J Diabetes Dev Ctries.

[26] De Sousa RAL, Improta-Caria AC, Aras-Júnior R (2021). Physical exercise effects on the brain during COVID-19 pandemic: links between mental and cardiovascular health. Neurol Sci.

[27] De Sousa RAL, Improta-Caria AC, Cassilhas RC (2021). Effects of physical exercise on memory in type 2 diabetes: a brief review. Metab Brain Dis.

[28] De Sousa RAL, Improta-Caria AC, de Jesus-Silva FM (2020). High-intensity resistance training induces changes in cognitive function, but not in locomotor activity or anxious behavior in rats induced to type 2 diabetes. Physiol Behav.

[29] De Sousa RAL, Improta-Caria AC, de Souza BS, F,  (2021). Exercise-linked irisin: consequences on mental and cardiovascular health in type 2 diabetes. Int J Mol Sci.

[30] De Sousa RAL, Mendes BF, Costa-Pereira L (2022). Accumulated high-intensity interval training protocol: a new approach to study health markers in wistar rats. J Vis Exp.

[31] De Sousa RAL, Peixoto MFD, Leite HR (2020). Neurological consequences of exercise during prenatal Zika virus exposure to mice pups. Int J Neurosci.

[32] De Sousa RAL, Rocha-Dias I, de Oliveira LRS (2021). Molecular mechanisms of physical exercise on depression in the elderly: a systematic review. Mol Biol Rep.

[33] De Sousa RAL, Santos LG, Lopes PM (2021). Physical exercise consequences on memory in obesity: a systematic review. Obes Rev.

[34] Deng J, Zhou F, Hou W (2021). The prevalence of depression, anxiety, and sleep disturbances in COVID-19 patients: a meta-analysis. Ann NY Acad Sci.

[35] Liegro Di, Schiera P, Liegro Di (2019). Physical activity and brain health. Genes.

[36] Di Stefano V, Ornello R, Gagliardo A (2021). Social distancing in chronic migraine during the covid-19 outbreak: results from a multicenter observational study. Nutrients.

[37] Dinel AL, André C, Aubert A (2011). Cognitive and emotional alterations are related to hippocampal inflammation in a mouse model of metabolic syndrome. PLoS ONE.

[38] Douaud G, Lee S, Alfaro-Almagro F (2022). SARS-CoV-2 is associated with changes in brain structure in UK biobank. Nature.

[39] Elmquist JK, Sleiman SF, Henry J (2016). Exercise promotes the expression of brain derived neurotrophic factor (BDNF) through the action of the ketone body b-hydroxybutyrate. Elife.

[40] Fajgenbaum DC, June CH (2020). Cytokine storm. N Engl J Med.

[41] Fernandez-Ruiz R, Paredes JL, Niewold TB (2021). COVID-19 in patients with systemic lupus erythematosus: lessons learned from the inflammatory disease. Transl Res.

[42] Fernández RS, Crivelli L, Guimet NM (2022). Psychological distress and mental health trajectories during the COVID-19 pandemic in Argentina: a longitudinal study. Sci Rep.

[43] Garbarino S, Lanteri P, Bragazzi NL (2021). Role of sleep deprivation in immune-related disease risk and outcomes. Commun Biol.

[44] Garber CE, Blissmer B, Deschenes MR (2011). Quantity and quality of exercise for developing and maintaining cardiorespiratory, musculoskeletal, and neuromotor fitness in apparently healthy adults: guidance for prescribing exercise. Med Sci Sports Exerc.

[45] Goldberger N, Bergman-Levy T, Haklai Z (2022). COVID-19 and severe mental illness in Israel: testing, infection, hospitalization, mortality and vaccination rates in a countrywide study. Mol Psychiatry.

[46] Gripp F, Gomes GDJ, De Sousa RAL (2022). A real-world high-intensity interval training protocol for cardiorespiratory fitness improvement. J Vis Exp.

[47] Guidon AC, Amato AA (2020). COVID-19 and neuromuscular disorders. Neurology.

[48] Hearon BA, Quatromoni PA, Mascoop JL, Otto MW (2014). The role of anxiety sensitivity in daily physical activity and eating behavior. Eat Behav.

[49] Improta-Caria AC, Aras MG, Nascimento L (2021). MicroRNAs regulating renin-angiotensin-aldosterone system, sympathetic nervous system and left ventricular hypertrophy in systemic arterial hypertension. Biomolecules.

[50] Improta-Caria AC, Soci ÚPR, Pinho CS (2021). Physical exercise and immune system: perspectives on the COVID-19 pandemic. Rev Assoc Med Bras.

[51] Jodeiri Farshbaf M, Ghaedi K, Megraw TL (2016). Does PGC1α/FNDC5/BDNF elicit the beneficial effects of exercise on neurodegenerative disorders?. NeuroMolecular Med.

[52] Júnior RA, Durães A, Roever L (2021). The impact of COVID-19 on the cardiovascular system. Rev Assoc Med Bras.

[53] Kalita J, Tripathi A, Dongre N, Misra UK (2021). Impact of COVID-19 pandemic and lockdown in a cohort of myasthenia gravis patients in India. Clin Neurol Neurosurg.

[54] Kim TW, Baek KW, Yu HS (2020). High-intensity exercise improves cognitive function and hippocampal brain-derived neurotrophic factor expression in obese mice maintained on high-fat diet. J Exerc Rehabil.

[55] Kissler SM, Tedijanto C, Goldstein E (2020). Projecting the transmission dynamics of SARS-CoV-2 through the postpandemic period. Science.

[56] Kraemer MUG, Yang CH, Gutierrez B (2020). The effect of human mobility and control measures on the COVID-19 epidemic in China. Science.

[57] Krishnan V, Nestler EJ (2008). The molecular neurobiology of depression. Nature.

[58] Lam K, Lee JH, Cheng P (2021). Pediatric stroke associated with a sedentary lifestyle during the SARS-CoV-2 (COVID-19) pandemic: a case report on a 17-year-old. Neurol Sci.

[59] Leoni de Sousa RA (2019). Cross-talk between obesity and central nervous system: role in cognitive function. Interv Obes Diabetes.

[60] Liu W, Ge T, Leng Y (2017). The role of neural plasticity in depression: from hippocampus to prefrontal cortex. Neural Plast.

[61] Mao L, Jin H, Wang M (2020). Neurologic manifestations of hospitalized patients with coronavirus disease 2019 in Wuhan, China. JAMA Neurol.

[62] Mariani N, Cattane N, Pariante C, Cattaneo A (2021). Gene expression studies in depression development and treatment: an overview of the underlying molecular mechanisms and biological processes to identify biomarkers. Transl Psychiatry.

[63] Mekkawy DA, Hamdy S, Abdel-Naseer M (2022). Neurological manifestations in a cohort of Egyptian patients with COVID-19: a prospective, multicenter, observational study. Brain Sci.

[64] Morovatdar N, Di Napoli M, Stranges S (2021). Regular physical activity postpones age of occurrence of first-ever stroke and improves long-term outcomes. Neurol Sci.

[65] Nami M, Mehrabi S, Kamali AM (2020). A new hypothesis on anxiety, sleep insufficiency, and viral infections; reciprocal links to consider in today’s “world vs. COVID-19” endeavors. Front Psychiatry.

[66] Ng TKY, Kwok CKC, Ngan GYK (2022). Differential effects of the COVID-19 pandemic on physical activity involvements and exercise habits in people with and without chronic diseases: a systematic review and meta-analysis. Arch Phys Med Rehabil.

[67] Ostler JE, Maurya SK, Dials J (2014). Effects of insulin resistance on skeletal muscle growth and exercise capacity in type 2 diabetic mouse models. Am J Physiol Endocrinol Metab.

[68] Pandey K, Thurman M, Johnson SD (2021). Mental health issues during and after COVID-19 vaccine era. Brain Res Bull.

[69] Pedersen BK, Saltin B (2015). Exercise as medicine—evidence for prescribing exercise as therapy in 26 different chronic diseases. Scand J Med Sci Sport.

[70] Pfefferbaum B, North CS (2020). Mental health and the Covid-19 pandemic. N Engl J Med.

[71] Qaisar R, Bhaskaran S, Van Remmen H (2016). Muscle fiber type diversification during exercise and regeneration. Free Radic Biol Med.

[72] Qiao X, Gai H, Su R (2018). PI3K-AKT-GSK3β-CREB signaling pathway regulates anxiety-like behavior in rats following alcohol withdrawal. J Affect Disord.

[73] Raony Í, de Figueiredo CS, Pandolfo P (2020). Psycho-Neuroendocrine-immune interactions in COVID-19: potential impacts on mental health. Front Immunol.

[74] Salari N, Hosseinian-Far A, Jalali R (2020). Prevalence of stress, anxiety, depression among the general population during the COVID-19 pandemic: a systematic review and meta-analysis. Global Health.

[75] Soares FHR, de Sousa MBC (2013). Different types of physical activity on inflammatory biomarkers in women with or without metabolic disorders: a systematic review. Women Heal.

[76] de Sousa RAL, Hagenbeck KF, Arsa G, Pardono E (2020). Moderate/high resistance exercise is better to reduce blood glucose and blood pressure in middle-aged diabetic subjects. Rev Bras Educ Física e Esporte.

[77] De Sousa RAL, de Lima NS, Amorim FT (2021). Endurance and high-intensity interval training improve the levels of anxiety and quality of life in overweight men. Rev Assoc Med Bras.

[78] Strawbridge R, Young AH, Cleare AJ (2017). Biomarkers for depression: recent insights, current challenges and future prospects. Neuropsychiatr Dis Treat.

[79] Tibubos AN, Otten D, Ernst M, Beutel ME (2021). A systematic review on sex- and gender-sensitive research in public mental health during the first wave of the COVID-19 crisis. Front Psychiatry.

[80] Tu WJ, Qiu HC, Liu Q (2018). Decreased level of irisin, a skeletal muscle cell-derived myokine, is associated with post-stroke depression in the ischemic stroke population. J Neuroinflamm.

[81] Vaduganathan M, Vardeny O, Michel T (2020). Renin-angiotensin-aldosterone system inhibitors in patients with Covid-19. N Engl J Med.

[82] Van Sloten TT, Schram MT, Adriaanse MC (2014). Endothelial dysfunction is associated with a greater depressive symptom score in a general elderly population: the Hoorn study. Psychol Med.

[83] Vancini RL, de Lira CAB, Anceschi SA (2018). Anxiety, depression symptoms, and physical activity levels of eutrophic and excess-weight Brazilian elite police officers: a preliminary study. Psychol Res Behav Manag.

[84] Wang LR, Baek SS (2018). Treadmill exercise activates PI3K/Akt signaling pathway leading to GSK-3β inhibition in the social isolated rat pups. J Exerc Rehabil.

[85] Willey B, Mimmack K, Gagliardi G (2022). Racial and socioeconomic status differences in stress, posttraumatic growth, and mental health in an older adult cohort during the COVID-19 pandemic. eClinicalMedicine.

[86] World Health Organization (2017). Depression and other common mental disorders global health estimates.

[87] Wrann CD, White JP, Salogiannnis J (2013). Exercise induces hippocampal BDNF through a PGC-1α/FNDC5 pathway. Cell Metab.

[88] Yachou Y, El Idrissi A, Belapasov V, Ait Benali S (2020). Neuroinvasion, neurotropic, and neuroinflammatory events of SARS-CoV-2: understanding the neurological manifestations in COVID-19 patients. Neurol Sci.

[89] Yang T, Nie Z, Shu H (2020). The role of BDNF on neural plasticity in depression. Front Cell Neurosci.

[90] Zhang SX, Wang Y, Rauch A, Wei F (2020). Unprecedented disruption of lives and work: Health, distress and life satisfaction of working adults in China one month into the COVID-19 outbreak. Psychiatry Res.

[91] Zubair AS, McAlpine LS, Gardin T (2020). Neuropathogenesis and neurologic manifestations of the coronaviruses in the age of coronavirus disease 2019: a review. JAMA Neurol.

